# Transcriptome analysis reveals ethylene-mediated defense responses to *Fusarium oxysporum* f. sp. *cucumerinum* infection in *Cucumis sativus* L.

**DOI:** 10.1186/s12870-020-02537-7

**Published:** 2020-07-16

**Authors:** Jingping Dong, Yuean Wang, Qianqian Xian, Xuehao Chen, Jun Xu

**Affiliations:** 1 School of Horticulture and Plant Protection, Yangzhou University, Yangzhou, Jiangsu Province China; 2 State Key Laboratory of Vegetable Germplasm Innovation, Tianjin, China

**Keywords:** Cucumber, Exogenous ethylene, *Fusarium* wilt, RNA-seq

## Abstract

**Background:**

*Fusarium* wilt, caused by *Fusarium oxysporum* f. sp. *cucumerinum* (Foc), is a severe disease affecting cucumber (*Cucumis sativus* L.) production worldwide, but mechanisms underlying *Fusarium* wilt resistance in cucumber remain unknown. To better understand of the defense mechanisms elicited in response to Foc inoculation, RNA sequencing-based transcriptomic profiling of responses of the *Fusarium* wilt-resistant cucumber line ‘Rijiecheng’ at 0, 24, 48, 96, and 192 h after Foc inoculation was performed.

**Results:**

We identified 4116 genes that were differentially expressed between 0 h and other time points after inoculation. All ethylene-related and pathogenesis-related genes from the differentially expressed genes were filtered out. Real-time PCR analysis showed that ethylene-related genes were induced in response to Foc infection. Importantly, after Foc infection and exogenous application of ethephon, a donor of ethylene, the ethylene-related genes were highly expressed. In response to exogenous ethephon treatment in conjunction with Foc inoculation, the infection resistance of cucumber seedlings was enhanced and endogenous ethylene biosynthesis increased dramatically.

**Conclusion:**

Collectively, ethylene signaling pathways play a positive role in regulating the defense response of cucumber to Foc infection. The results provide insight into the cucumber *Fusarium* wilt defense mechanisms and provide valuable information for breeding new cucumber cultivars with enhanced *Fusarium* wilt tolerance.

## Background

Cucumber is among the most widely cultivated vegetables in the world. Given the continuous cropping systems used in cucumber production in China, cucumber *Fusarium* wilt (FW) incidence is aggravated year-on-year. FW is a typical soil-borne disease caused by *Fusarium oxysporum* f. sp. *cucumerinum* (Foc) [1], which usually results in severe losses in global cucumber output [2]. Foc includes four races, designated races 1, 2, 3, and 4, that are prevalent mainly in the United States, Israel, Japan, and China [3–5], respectively. Foc can survive in soil and seeds for many years or even decades [6]. Hyphae of this pathogen penetrate cucumber roots, then spread to the vascular tissues and occludes the xylem vessels, and also produces a toxin to kill cells, which leads to wilting of the leaves or even the entire plant, until plant death occurs several days or weeks after infection [7, 8]. FW is extremely difficult to be controlled and changes in the pathogenicity of Foc have led to the ineffectiveness of certain fungicides [9, 10]. Therefore, identification of disease resistance genes, augmentation of germplasm pools, and breeding FW-resistant cucumber cultivars is an efficient strategy to control cucumber FW [11].

Increasing research attention is focused on the potential of functional genomics to study the host immune system to enhance disease protection [12]. The defense mechanisms induced in a plant upon exposure to a pathogen, including the network of cross-communicating signaling pathways, may halt pathogen infection. Ethylene (ET) is the principal mediator in plants of these signaling pathways [13, 14]. ET was first identified phytohormone owing to its effects on fruit maturation, senescence, germination, cell elongation, and flowering [15, 16], and was subsequently determined to function as a modulator of the plant immune signaling network [17]. Previous molecular studies indicate that in plants many diverse plant hormone-regulated pathways involved in resistance to biotic stresses are triggered through biosynthesis of effector proteins by the pathogen [18]. The predominant symptom elicited by Foc infection of cucumber is wilting, which is caused by disturbance of the water balance [19, 20]. Abscisic acid (ABA) might play a crucial regulatory role in modifying stomatal behavior, which has a major impact on disturbance of water loss in cucumber [21]. Disruption of the transcription factor AtMYC2, which is a positive regulator of ABA signaling [22], induces expression of ethylene-responsive genes [23]. In *Arabidopsis*, ethylene response 1 (ETR1), which belongs to the ET receptor family, positively influences pathogen-mediated responses [24]. It is also known that ET signaling pathways play a crucial role in the inducible plant defense response [18, 25]. When soybean was infected with *Soybean mosaic virus* in the shade, ET signaling was activated, which might play an important role in regulating the defense response [26]. Liu et al. [27] observed that ET signaling pathways might positively regulate the defense response of *Panax notoginseng* to *Fusarium solani*.

In this research, we conducted a comprehensive and global analysis of the transcriptome during the defense response of cucumber to Foc infection using a high-throughput RNA sequencing (RNA-seq) approach. We aimed to identify differentially expressed genes (DEGs) and enriched pathways that contribute to disease resistance after inoculation of cucumber roots with spore suspensions at different time points. The findings provide insight into ET-related candidate genes and pathways associated with resistance to FW, which will contribute to broader knowledge of the resistance mechanism in cucumber to Foc.

## Results

### Quality analysis of RNA-seq data from cucumber roots infected by Foc

Roots of cucumber ‘Rijiecheng’, the Foc-resistant line, were sampled with three biological replicates and sequenced for transcriptome analysis. The Pearson’s Correlation Coefficient (r) was used as the evaluation index of the repetitive correlation. After quality checks of raw reads, more than 100.75 Gb clean data were obtained from 15 RNA-seq libraries (Additional file 1: Table S1; deposited in the NCBI SRA database under accession No. PRJNA472169). The number of reads mapped to the cucumber Chinese Long reference genome exceeded 29.54 million (70.64% of the total reads). For each sample, the Q30 value was higher than 85.27%, indicating that the sequence data were of high quality.

### Identification of DEGs and expression profiles of ET-related genes

**Fig. 1:**
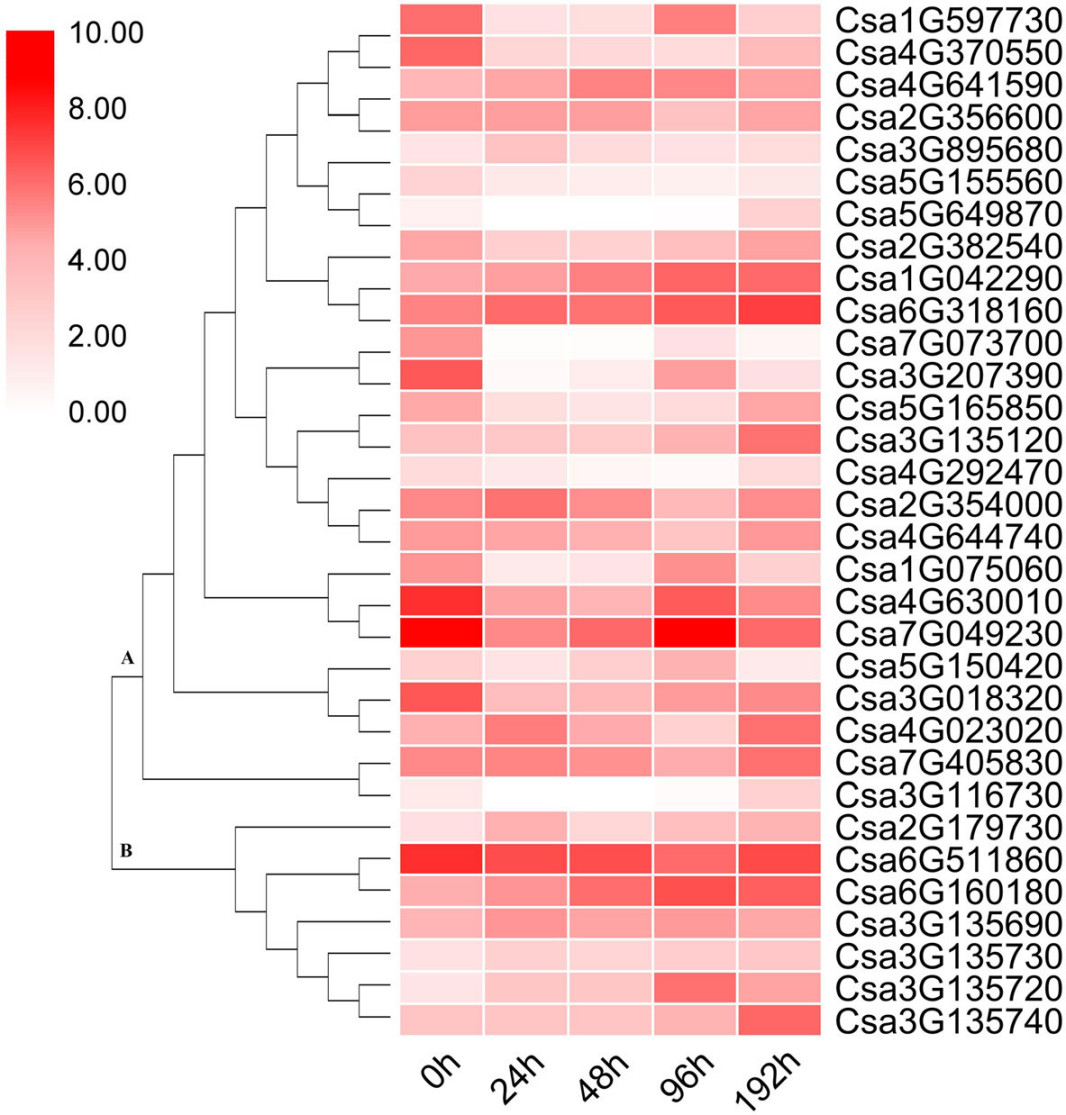
Hierarchical clustering of ethylene- related DEGs. Fold- difference was designated as fragments per kilobase of transcript per million fragments mapped. The maximum value was ‘1’ for each gene. The darker the shade of red, the higher the expression level. 0 h, 24 h, 48 h, 96 h, and 192 h indicate the sampling time points after inoculation

**Fig. 2:**
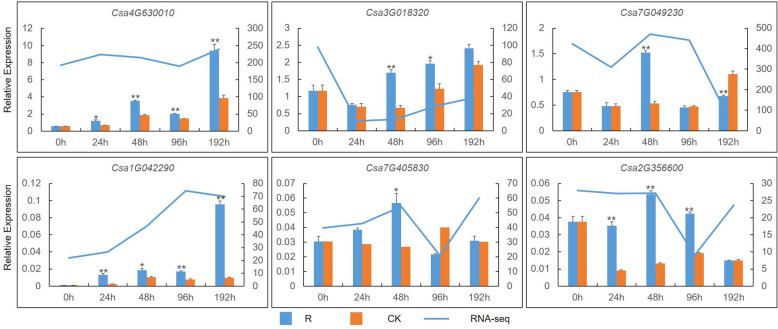
Changes in relative expression level of six DEGs analyzed by qPCR and RNA-seq. The left vertical axis indicates the relative expression level determined by qPCR, the right vertical axis indicates the FPKM value determined by RNA-seq. 0 h, 24 h, 48 h, 96 h, and 192 h indicate time points after Foc inoculation. The entire cucumber roots were the samples. R, ‘Rijiecheng’, a Foc-resistant cucumber line, inoculated with Foc; CK, ‘Rijiecheng’ inoculated with sterile water. At least three biological replicates were performed in each experiment group. * *P* < 0.05, ** *P* < 0.01

Using fold change ≥1 and false discovery rate (FDR) < 0.01 as cutoff, 4116 DEGs were identified. Among these genes, 1469 genes were upregulated and 2647 genes were downregulated (Additional file 2: Figure S1). Thirty-two ET-related DEGs were filtered out (Fig. 1). Of the filtered genes, group A consisted of ET-responsive transcription factors including 25 DEGs, and group B comprised 1-aminocyclopropane-1-carboxylate (ACC) oxidase, which catalyzes the oxidative cleavage of ACC to form ET. Group A was divided into several subgroups and the expression profiles of DEGs in each subgroup was similar. Quantitative real-time PCR (qPCR) analysis was used to verify the DEGs by selection of six DEGs that showed a high relative expression level and upregulated expression (Fig. 2). The expression trends were consistent across both qPCR and RNA-seq analysis, which indicated the relative expression of these genes between the two methods showed a correlation.

### ET affects growth of Foc

**Fig. 3:**
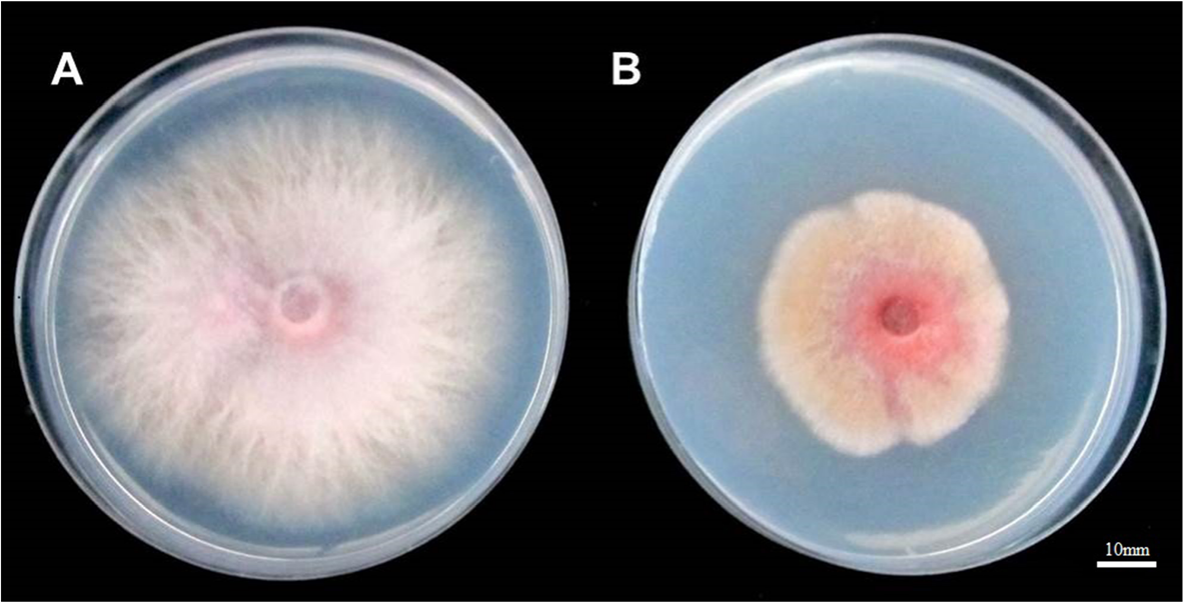
The phenotypic differences in vitro of Foc with ethephon. In vitro phenotypic differences between the Foc strain cultured on PDA medium supplemented with (**a**) sterile water and (**b**) 1000 ppm ethephon

To clarify whether ET affected the growth of Foc, we cultured Foc on potato dextrose agar (PDA) medium supplemented with 1000 ppm ethephon. Mycelial growth of the Foc strain on PDA supplemented with sterile water (the control) was superior to that on PDA with ethephon (Fig. 3). The average diameter of mycelial colonies on PDA supplemented with sterile water was 74.33 mm, whereas the average diameter on PDA supplemented with ethephon was 43.67 mm. The color of mycelia was darker on PDA supplemented with ethephon than that on the control medium. These results indicated that ET could suppress the growth of Foc in the presence of 1000 ppm ethephon.

### ET increases resistance of cucumber seedlings to Foc

**Fig. 4:**
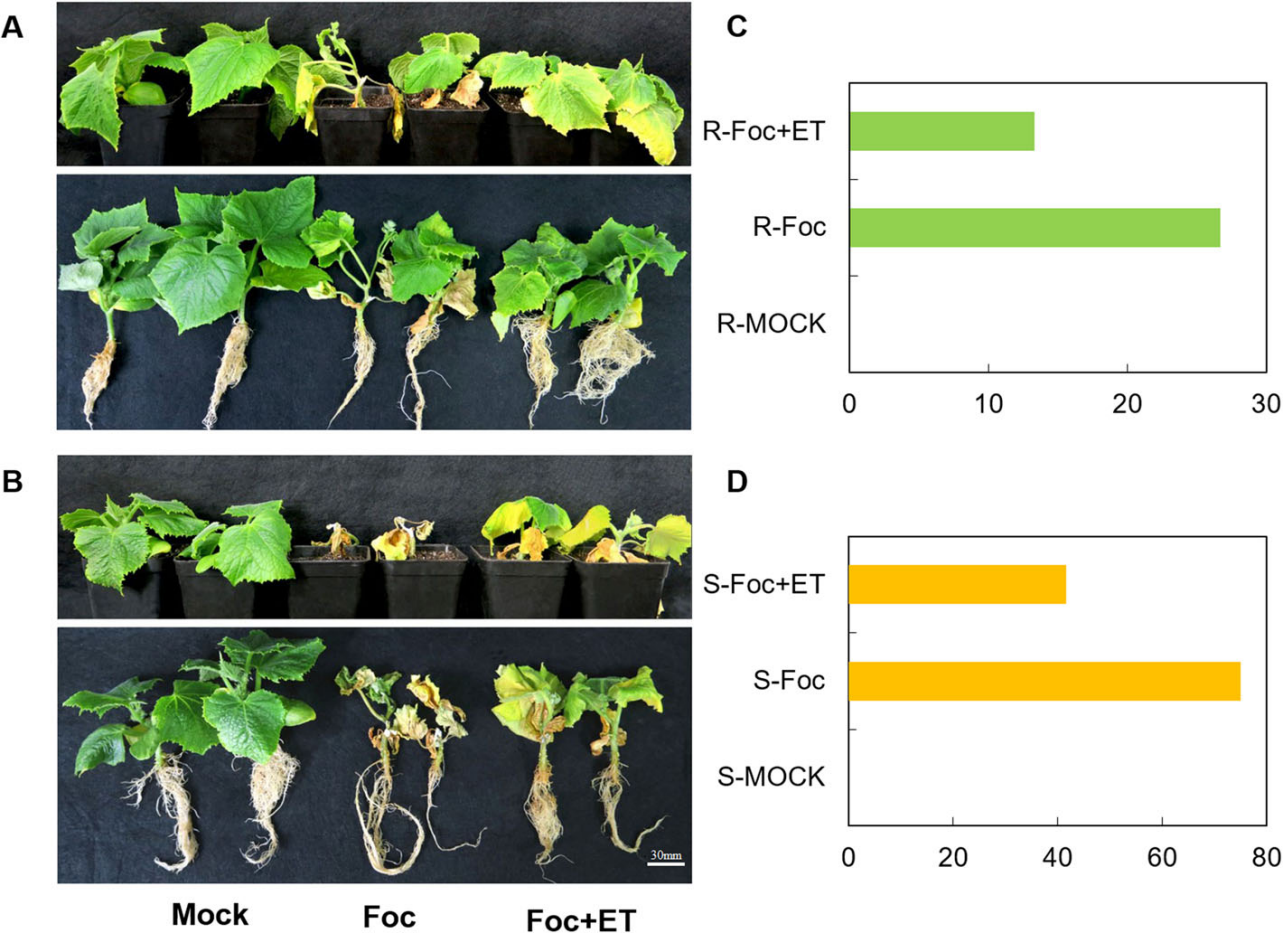
Phenotypic differences and disease index of cucumber seedlings. **a**, **b** Phenotypic differences of cucumber seedlings. The one third of seedlings roots treated with mock solutions, one third were inoculated with a Foc strain (spore concentration 10^6^ conidia/mL), and the others were inoculated with Foc and sprayed with exogenous ethephon on leaves (concentration 10 ppm, Foc + ET) at the same time. **c**, **d** Disease index for the phenotypes of the cucumber seedlings. The Y-axis indicates the disease index. The typical phenotypic differences and disease index were recorded and calculated 3 weeks after treatment. R, ‘Rijiecheng’ (Foc-resistant line), S, ‘Superina’, (Foc-sensitive line)

To test the possibility that ET may enhance the resistance response in cucumber seedlings, cucumber seedlings at the second-true-leaf stage were inoculated with Foc and exogenous ethephon and grown for 3 weeks. The disease grade was numbered and disease index was calculated. Four days after inoculation, cotyledons of the Foc-sensitive cultivar ‘Superina’ cultured with Foc began to turn yellow, whereas the same symptoms of Superina seedlings cultured with Foc and ethephon were observed at 10 days after inoculation. Symptoms in seedlings of the Foc-resistant cultivar ‘Rijiecheng’ were observed at 7 days after inoculation with Foc, and at 15 days after inoculation with Foc and exogenous ethephon. The disease indices of Superina under Foc inoculation and Foc inoculation in conjunction with exogenous ethephon were 75 and 41.67, respectively. The Rijiecheng’s were 26.67 and 13.33, respectively. The disease indices of two cultivars under Foc inoculation were higher than those under Foc inoculation in conjunction with exogenous ethephon (Fig. 4c, d). Thus, the resistance of cucumber seedlings inoculated with Foc and ET was superior to that of seedlings inoculated only with Foc (Fig. 4a, b**)** in both a resistant cultivar (Rijiecheng) and a sensitive cultivar (Superina). These results indicated that ET can increase the resistance of cucumber seedlings to Foc.

### Expression profiles of ET-related genes in response to exogenous ET and Foc infection

**Fig. 5:**
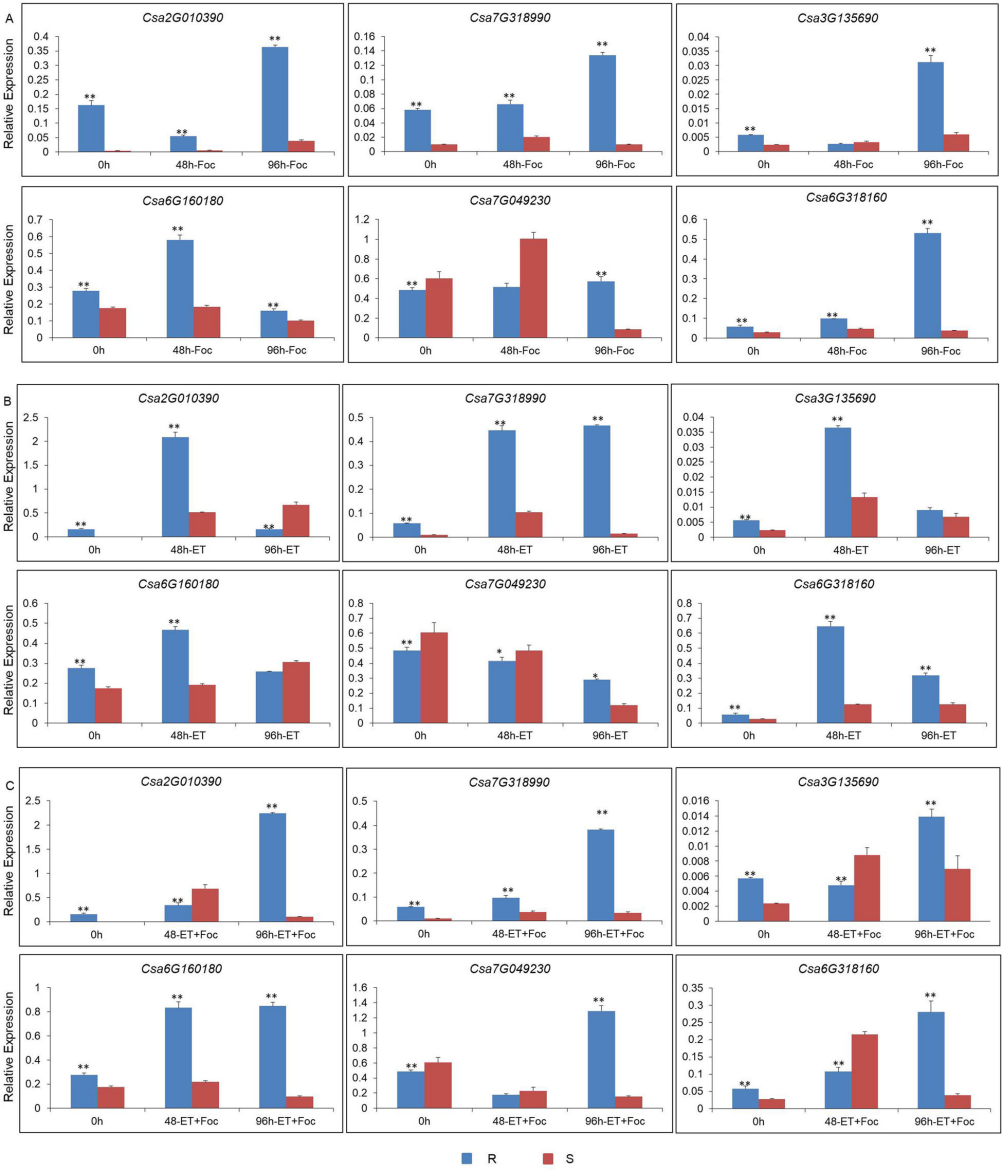
Expression profiles of six candidate ethylene- related genes. These genes were calculated in cucumber at 0 h, 48 h, and 96 h (**a**) inoculation with Foc, **b** spray treatment with exogenous ET, and (**c**) after inoculation with Foc and spray treatment with exogenous ET. R, ‘Rijiecheng’ (Foc-resistant line), S, ‘Superina’ (Foc-sensitive line)

Fourteen genes that showed high relative expression levels were selected to confirm the expression levels in response to exogenous ethephon using the qPCR method. *Csa2G010390* and *Csa7G318990* encoded pathogenesis-related (PR) proteins, *Csa4G630010*, *Csa7G049230*, *Csa2G382540*, *Csa1G042290*, *Csa4G641590*, *Csa2G354000*, and *Csa6G318160* encoded ET-responsive transcription factors, *Csa7G405830* encoded an ET receptor, and *Csa3G135690*, *Csa6G511860*, *Csa3G135740*, and *Csa6G160180* encoded ACC oxidases. Six of the 14 genes were chosen as representative genes in the ET pathway for cucumber resistance to Foc. The expression level of the two PR genes increased significantly in response to exogenous application of ET both in the Foc-resistant and -sensitive cultivars (Fig. 5a, b, c). The expression level was relatively higher in the Foc-resistant cultivar than that in the sensitive cultivar. Thus, the PR genes may be modulated by ET and the disease resistance of seedlings may be improved simultaneously. *Csa3G135690* and *Csa6G160180*, which encoding ACC oxidases, *Csa7G049230* and *Csa6G318160*, which encoding ET-responsive transcription factors, were upregulated by exogenous ethephon and Foc infection (Fig. 5c). The expression levels of the other 8 genes were shown in Additional file 3: Figure S2. All the primers were shown in Additional file 4: Table S2. In short, after inoculation with Foc, these ET signaling pathway genes were highly expressed in response to exogenous ethephon.

### Validation of endogenous ET of cucumber seedlings with exogenous ethephon and Foc infection

**Fig. 6:**
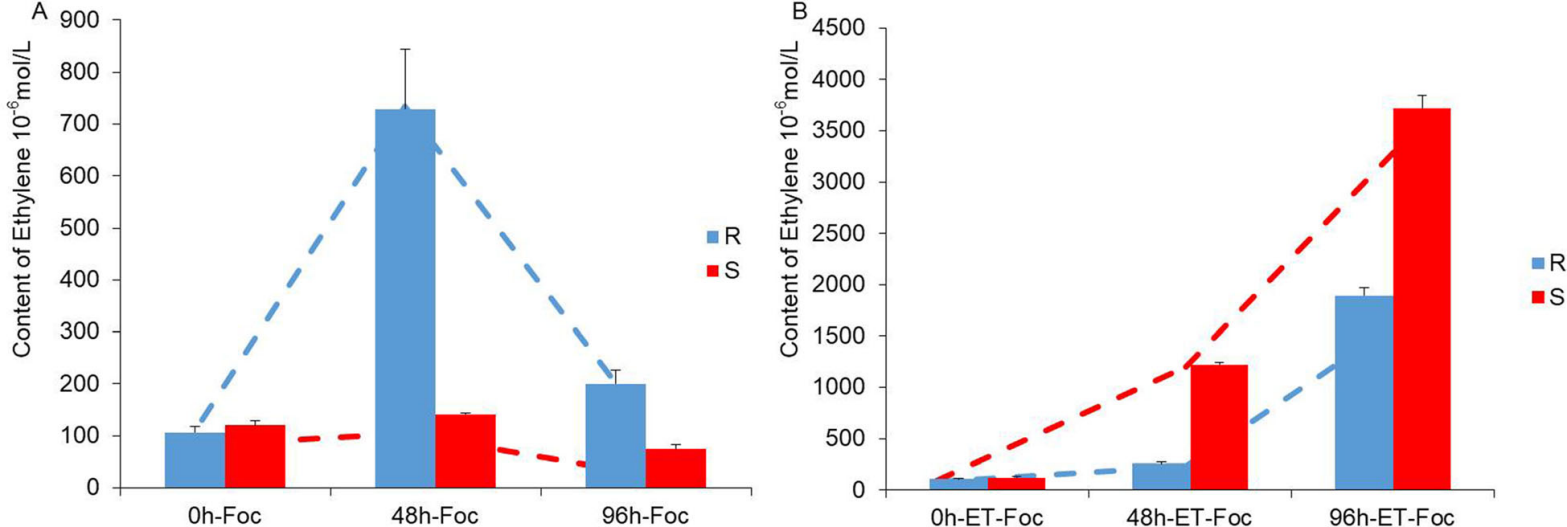
Endogenous ethylene production by the root of cucumber seedlings. These seedlings were treated at 0, 48, and 96 h (**a**) inoculation with Foc, **b** after inoculation with Foc and spray treatment with exogenous ET. R, ‘Rijiecheng’ (Foc-resistant line), S, ‘Superina’ (Foc-sensitive line)

Given that the ET-related genes were upregulated by Foc infection and exogenous application of ethephon, we measured the endogenous ET biosynthesis of cucumber seedlings in response to Foc inoculation and exogenous ethephon (Fig. 6a, b). The endogenous ethylene biosynthesis was higher in Rijiecheng than in Superina at 48 and 96 h in response to Foc infection alone. The endogenous ethylene concentration was about four-times higher at 48 h and twice as high at 96 h in Superina than in Rijiecheng in response to exogenous ethephon and Foc infection. Endogenous ET biosynthesis increased markedly after treatment with exogenous ethephon. These experiments indicated that Foc may induce the biosynthesis of endogenous ET in cucumber seedlings and exogenous ethephon may enhance this response.

## Discussion

As a common soil-borne disease, FW presents a serious barrier to continuous cropping of cucumber [28]. There is currently no effective measure to control this disease except for breeding resistant cultivars. Exploration of genes associated with pathogen resistance in plants using diverse approaches is a major research focus. The evolution of resistance genes has been observed in numerous model plants [29–31]. Expression profiling of genes related to FW resistance in cucumber has not previously been reported. In the present study, we gained insight into the genes associated with resistance to Foc in cucumber based on RNA-seq analysis of the global transcriptome profile in cucumber at different time points after inoculation. Global data analyses can help in elucidating genes related resistance and screening the key genes that control resistance to FW in cucumber, finally.

The whole-genome transcriptome analysis revealed that ET-responsive genes were distinctly induced in response to Foc infection. Thus, ET signaling was involved in the interaction between cucumber and Foc, and genes encoding ET receptors were differentially expressed before and after inoculation. In previous studies, ET signaling pathways under physiological stresses or other abiotic stresses were often reported. The present experiments suggest that ET is a crucial factor in cucumber resistance to Foc. Similar findings were reported for *Medicago truncatula* infected by *Rhizoctonia solani* [32], which led to upregulation of ET signaling. ET receptors are downstream regulatory factors of the ET-signaling pathway [25], which regulate plant growth, development, and many types of stress response [33]. In tomato, *Trichoderma harzianum* infection induced the expression of PR proteins, which were markers of the ET-dependent signaling pathways [29]. Similarly, two PR genes were upregulated by ET in the present study. PR genes perform a pioneering role in the diagnosis of plant immunity under pathogen challenge. Expression of genes that encode PR proteins appear to be upregulated rapidly in response to pathogen infection. *PR-1* was detected in pathogen-stressed *Nicotiana tabacum* plants [34]. These PR proteins are an important component of plant resistance to pathogens [35]. Ohme-Takagi and Shinishi [36] reported that the GCC box of PR genes promoted the functions of an ET-responsive element [25]. Enhanced disease resistance 1 (EDR1) encodes a CTR1-like kinase [37], which is a negative regulator of ET responses [38, 39]. Tang et al. [40] considered that EDR1 may function in ET signaling to promote cell death.

Plant immune systems may also be activated by exogenous application of relevant molecules to modulate the defense response. Phytohormones regulate internal as well as external environmental signals [41]. The ethephon had the capacity to synthesize endogenous ethylene in transgenic apple fruit [42]. The present study revealed that endogenous ET biosynthesis is significantly elevated with exogenous application of ethephon. Exogenous ethephon was applied to verify that ET may enhance the resistance of cucumber to Foc. Treatments with exogenous ethylene provide a safe and effective method for control of pear browning [43]. Exogenous application of ACC, the precursor of ET, enhanced the resistance of *NbALD1*-transgenic plants of *Nicotiana benthamiana* to *Turnip mosaic virus* [44].

The present transcriptome data provided an improved understanding of gene expression profiles in cucumber upon infection by Foc. The data provide a comprehensive overview of the functions and effects of ET-related genes involved in the cucumber defense response. ET-related genes were induced in response to Foc inoculation; ET-related and PR genes were highly expressed in response to exogenous ethephon application, and the disease resistance of cucumber seedlings was enhanced simultaneously. The present results provide a foundation for further discovery of gene functions in cucumber. Future research should focus on overexpression and knockout of candidate genes that enhance resistance to Foc and elucidate the molecular mechanisms of resistance in cucumber.

## Conclusions

By means of a global transcriptome analysis, we identified 4116 genes that were differentially expressed between 0 h and 24, 48, 96, and 192 h after inoculation with Foc. In response to Foc infection and exogenous application of ethephon, ET-related and PR genes were confirmed to be highly expressed using qPCR analysis. Exogenous ethephon treatment together with Foc inoculation enhanced the disease resistance of cucumber seedlings and endogenous ET biosynthesis was substantially increased. The present results illustrate that ET signaling pathways play a role in positively regulating the defense response of cucumber to Foc. The findings will be helpful for elucidating the cucumber FW defense mechanisms, and information on the candidate genes will enrich the breeding of new cucumber cultivars with enhanced FW tolerance.

## Methods

### Fungal culture and plant materials

The Foc fungal strain was isolated from the experimental field of Yangzhou University, Jiangsu Province, China, and propagated on PDA plates at 28 °C for 4 days, then cultured in potato dextrose broth on a shaker at 180 rpm at 28 °C for 3 days. The spore suspension was diluted to 1 × 10^6^ spores per milliliter with sterile distilled water.

Seedlings of cucumber ‘Rijiecheng’ and ‘Superina’, moderately Foc-resistant cultivar and Foc-sensitive cultivar, were grown in 32-well plates filled with an aseptic organic substrate (contents of total nitrogen, phosphorus, and potassium = 40–60 g/kg, content of humus ≥350 g/kg, pH = 6.5–7.5) at 25 °C/18 °C day/night temperatures with a 16 h/8 h photoperiod. Seedlings were infected with Foc by irrigation of the roots with a fungal spore suspension (5 mL per seedling) at the second-true-leaf stage. Cucumber roots were sampled at 0, 24, 48, 96, and 192 h after inoculation with three biological replicates. All the seeds were obtained from the homozygous inbred lines of our own laboratory.

### RNA extraction, cDNA library construction, Illumina sequencing, and analysis of sequence reads

Total RNA was isolated using the TaKaRa MiniBEST Plant RNA Extraction Kit (TaKaRa, China). The RNA concentration was measured using the Qubit RNA Assay Kit with a Qubit® 2.0 fluorometer (Life Technologies, USA). One microgram of RNA was used as input for the RNA sample preparations. The mRNA was purified from the total RNA using poly-T oligo-attached magnetic beads. Fragmentation was carried out using divalent cations under an elevated temperature in NEBNext® First Strand Synthesis Reaction Buffer (5×). The first-strand cDNA was synthesized using the mRNA fragments as templates. To select cDNA fragments of preferentially 200–250 bp in length, the library fragments were purified with the AMPure XP system (Beckman Coulter, USA). Eligible cDNAs were selected for PCR amplification, which was performed with Phusion® High-Fidelity DNA polymerase, Universal PCR primers, and Index (X) Primer. The PCR products were purified using the AMPure XP system and library quality was assessed with an Agilent Bioanalyzer 2100 system.

Primary cDNA produced using the Illumina HiSeq 2500 platform by BioMarker Technologies (Beijing, China) were termed raw reads. Clean reads were obtained by removing reads containing the adapter, reads containing poly-N, and low-quality reads. In addition, Q20, Q30, GC-content and sequence duplication values of the clean reads were calculated. The trimmed reads were aligned to the cucumber Chinese Long reference genome v2 [33], which was retrieved from http://cucurbitgenomics.org/organism/2. All downstream analyses were based on the clean, high-quality data. Quantification of gene expression levels was estimated as fragments per kilobase of transcript per million fragments mapped (FPKM) [45, 46] using Cufflinks (version: 2.1.1).

### Identification of DEGs and validation of RNA-seq by qPCR

We divided the data into four groups by comparing the data at 0 h with that at the other sampling time points. The analysis of DEGs for the four groups was performed using the DESeq R package (1.10.1) [47]. The *P* values were adjusted using the Benjamini–Hochberg approach for controlling the FDR. Genes with an adjusted FDR < 0.01 identified by DESeq and log_2_ FPKM (fold change) ≥ 1 were considered to be differentially expressed.

The root portion was placed as the expression samples. Total RNA of each condition was isolated according to the method part of Qi [48]. Primer sequences for qPCR were designed using Primer Premier 5. The qPCR analysis was performed using SYBR® Premix Ex Taq™ II (TaKaRa, China) in accordance with the manufacturer’s instructions. SYBR Green PCR cycling was performed on an Iqtm5 Multicolor qPCR detection system (Bio-Rad, USA) using 20 μL samples with the following temperature program: 95 °C for 3 min, followed by 39 cycles of 95 °C for 10 s, 60 °C for 20 s, and 72 °C for 20 s, then a melting curve analysis was performed. The relative quantification of gene expression was normalized to the tubulin alpha chain gene (*Csa4G000580*). Each condition of qPCR has three biological replicates.

### Evaluation of exogenous ethephon on Foc and cucumber seedlings

To ascertain the effect of ET on Foc growth, exogenous ethephon (a donor of ET) was incorporated in PDA medium. To 18 mL PDA medium was added either 2 mL ethephon (final concentration 1000 ppm) or 2 mL sterile water (as the control). Foc was cultured on the PDA medium at 25 °C for 5 days and the colony diameter was measured three times.

To examine the effect of ET on seedling resistance to Foc, one third of seedlings roots treated with mock solutions as the control, one third were inoculated with a Foc strain (spore concentration 10^6^ conidia per mL) using the root irrigation method, and the others were inoculated with Foc and sprayed with exogenous ethephon solution (concentration 10 ppm) on leaves at the same time. After 3 weeks, disease grades were recorded. The disease grades were divided into 0–4 grades, depending on the severity of the symptoms according to the method part of Dong [49]. The disease index was calculated using the following formula: Disease index (%) = $\frac{\sum \left(\mathrm{Disease}\ \mathrm{rating}\times \mathrm{Number}\ \mathrm{of}\ \mathrm{seedlings}\ \mathrm{with}\ \mathrm{disease}\ \mathrm{rating}\ \right)}{\mathrm{Total}\ \mathrm{number}\ \mathrm{of}\ \mathrm{seedlings}\times 4}$ × 100. Each experimental group included at least 15 individual seedlings and three biological replicates were treated. Mock inoculated seedlings were used as the control.

### Measurement of endogenous ET biosynthesis

The two cucumber inbred lines, ‘Rijiecheng’ and ‘Superina’, which are resistant and sensitive to Foc, respectively, were used in this experiment. Seedlings at the second-true-leaf stage were inoculated by irrigation with Foc (spore concentration 10^6^ conidia/mL) and sprayed with exogenous ethephon solution (concentration 10 ppm). Endogenous ET biosynthesis by the root portion of the seedlings was measured at 48 and 96 h after treatment. The root portion was placed in a sealed 30 mL ampoule for 24 h in the dark. For analysis of ET production, gas samples (1 mL) were collected using a syringe and injected into a gas chromatograph (Agilent 7890A, USA) fitted with a flame ionization and electron capture detectors [50]. Experiments were conducted with three biological replicates.

## Supplementary information

**Additional file 1: Table S1.** Data quality assessment of each sample.

**Additional file 2: Figure S1.** Differentially expressed genes (DEGs) at different time points after infection.

**Additional file 3: Figure S2.** Expression profiles of unselected candidate genes.

**Additional file 4: Table S2.** Primer sequences used for ethylene- related differentially expressed genes.

## Data Availability

All transcriptome data associated with this study have been submitted to the NCBI, and can be found using accession number PRJNA472169.

## Abbreviations

FW: *Fusarium* wilt
Foc: *Fusarium oxysporum* f. sp. *cucumerinum*
ET: Ethylene
ABA: Abscisic acid
ETR1: Ethylene response 1
RNA-seq: RNA sequencing
DEGs: Differentially expressed genes
FDR: False discovery rate
ACC: 1-aminocyclopropane-1-carboxylate
qPCR: Quantitative real-time PCR
PDA: Potato dextrose agar
PR: Pathogenesis-related
