# Supplementary material for: Transcriptome analysis reveals ethylene-mediated defense responses to Fusarium oxysporum f. sp. cucumerinum infection in Cucumis sativus L
Source: BMC Plant Biol. 2020 Jul 16;20:334. doi: 10.1186/s12870-020-02537-7 (PMC7364617; doi:10.1186/s12870-020-02537-7)
Supplement: Supplementary file 1 — Additional file 1: Table S1. Data quality assessment of each sample. [file 12870_2020_2537_MOESM1_ESM.pdf]

Additional file 1: Table S1. Data quality assessment of each sample.

Sample Name	Clean Reads	Total Reads	GC Content	%≥Q30	Reads aligned(%)
0h-1	22,248,742	44,497,484	44.38%	85.81%	73.16%
0h-2	20,558,568	41,117,136	43.77%	85.51%	73.78%
0h-3	25,880,302	45,007,340	43.71%	85.96%	73.19%
24h-1	21,797,728	43,595,456	44.09%	85.56%	73.41%
24h-2	21,472,350	42,944,700	43.78%	86.01%	73.70%
24h-3	25,019,013	50,038,026	44.79%	85.63%	71.99%
48h-1	20,348,174	40,696,348	44.25%	85.71%	72.59%
48h-2	23,102,884	46,205,768	44.10%	86.10%	73.27%
48h-3	21,121,583	42,243,166	44.15%	86.52%	74.47%
96h-1	21,766,632	43,533,264	44.52%	86.01%	73.23%
96h-2	23,102,884	43,851,420	44.24%	85.79%	73.34%
96h-3	24,339,657	48,679,314	45.14%	86.26%	72.26%
192h-1	26,070,671	52,141,342	44.84%	85.35%	71.41%
192h-2	25,880,302	51,760,604	45.18%	85.27%	70.64%
192h-3	22,887,881	45,775,762	44.49%	85.49%	72.02%

Sample Name (-1, -2, -3): three duplicate samples at each time point after inoculation.

%≥Q30: percentage of bases that the clean data quality value is greater than or equal to 30.
