# Supplementary material for: Transcriptome analysis reveals ethylene-mediated defense responses to Fusarium oxysporum f. sp. cucumerinum infection in Cucumis sativus L
Source: BMC Plant Biol. 2020 Jul 16;20:334. doi: 10.1186/s12870-020-02537-7 (PMC7364617; doi:10.1186/s12870-020-02537-7)
Supplement: Supplementary file 2 — Additional file 2: Figure S1. Differentially expressed genes (DEGs) at different time points after infection. [file 12870_2020_2537_MOESM2_ESM.pdf]

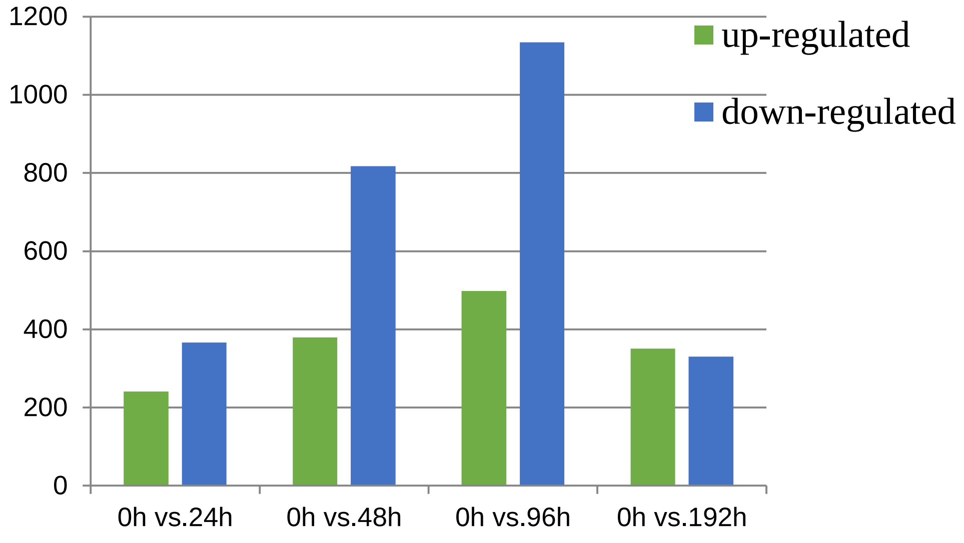

Additional file 2: Figure S1. Differentially expressed genes (DEGs) at different time points after infection. 0 h vs 24 h, 0 h vs 48 h, 0 h vs 96 h and 0 h vs 192 h (DEGs between 0 h and 24, 48, 96, and 192 h, respectively).
