# Supplementary material for: Transcriptome analysis reveals ethylene-mediated defense responses to Fusarium oxysporum f. sp. cucumerinum infection in Cucumis sativus L
Source: BMC Plant Biol. 2020 Jul 16;20:334. doi: 10.1186/s12870-020-02537-7 (PMC7364617; doi:10.1186/s12870-020-02537-7)
Supplement: Supplementary file 3 — Additional file 3: Figure S2. Expression profiles of unselected candidate genes. [file 12870_2020_2537_MOESM3_ESM.pdf]

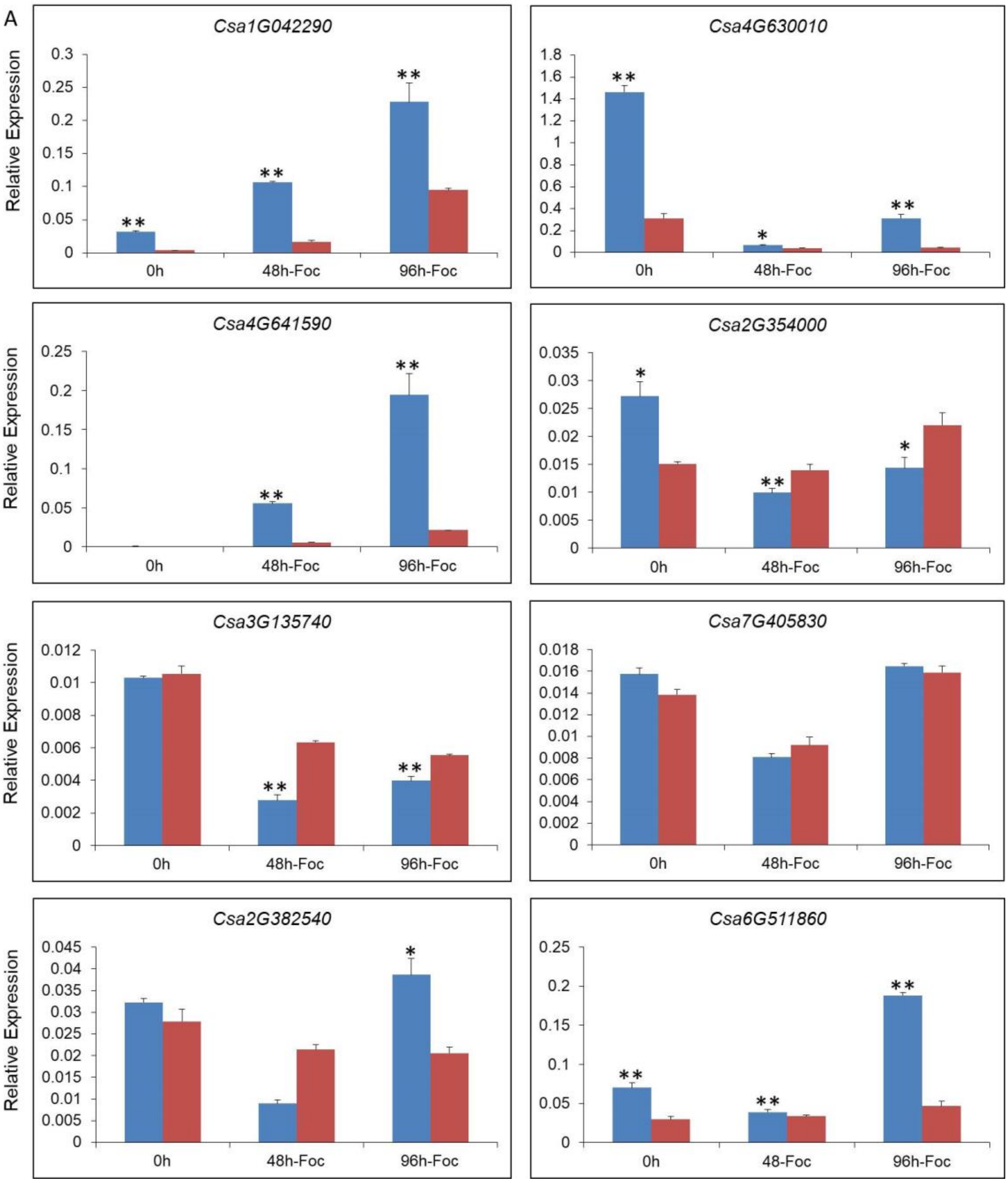

A
Csa1G042290
Csa4G630010
Csa4G641590
Csa2G354000
Csa3G135740
Csa7G405830
Csa2G382540
Csa6G511860
Relative Expression
0h
48h-Foc
96h-Foc
48-Foc

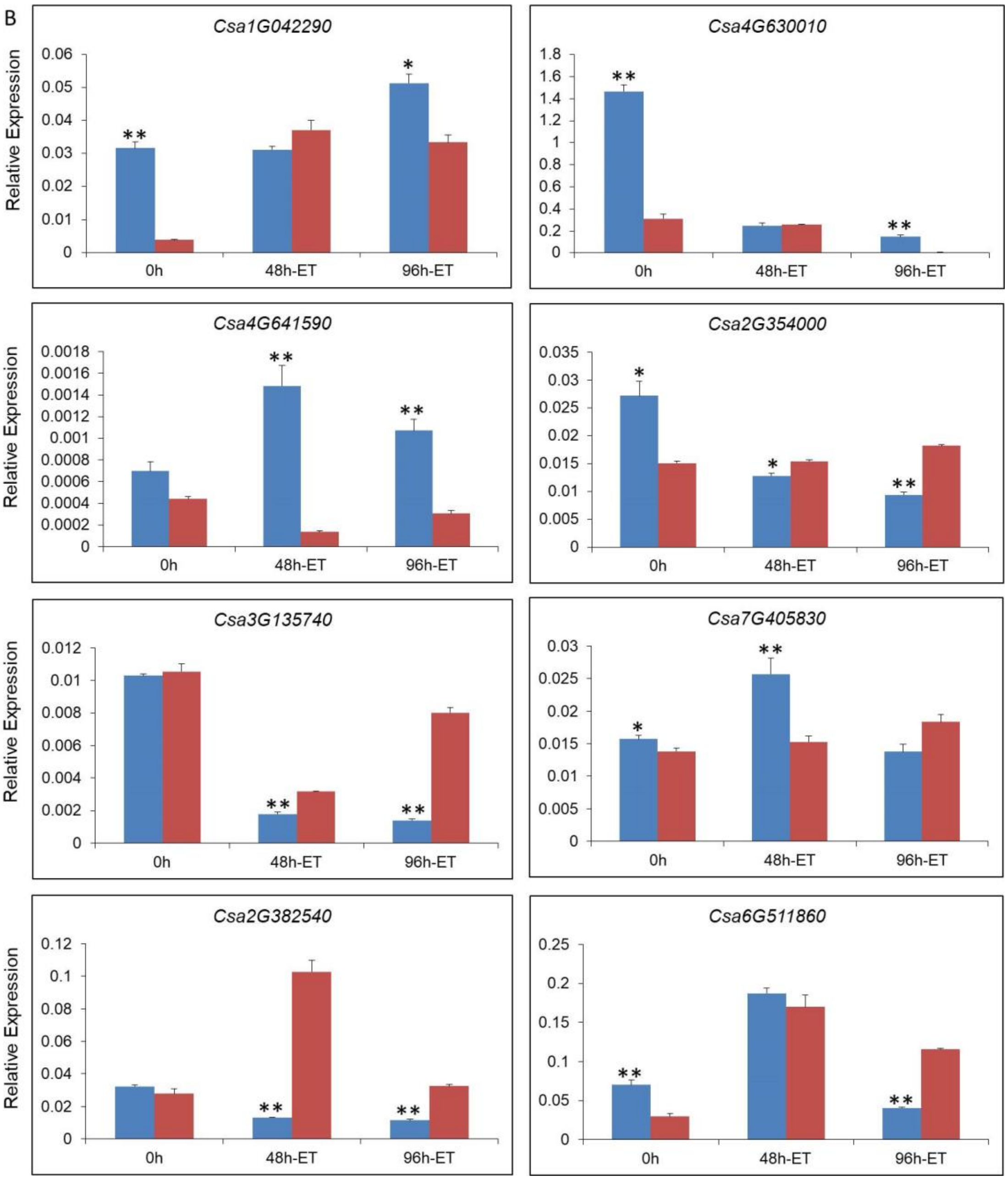
B
Csa1G042290
Relative Expression
0.06
0.05
0.04
0.03
0.02
0.01
0
0h
48h-ET
96h-ET
Csa4G630010
1.8
1.6
1.4
1.2
1
0.8
0.6
0.4
0.2
0
0h
48h-ET
96h-ET
Csa4G641590
Relative Expression
0.0018
0.0016
0.0014
0.0012
0.001
0.0008
0.0006
0.0004
0.0002
0
0h
48h-ET
96h-ET
Csa2G354000
0.035
0.03
0.025
0.02
0.015
0.01
0.005
0
0h
48h-ET
96h-ET
Csa3G135740
Relative Expression
0.012
0.01
0.008
0.006
0.004
0.002
0
0h
48h-ET
96h-ET
Csa7G405830
0.03
0.025
0.02
0.015
0.01
0.005
0
0h
48h-ET
96h-ET
Csa2G382540
Relative Expression
0.12
0.1
0.08
0.06
0.04
0.02
0
0h
48h-ET
96h-ET
Csa6G511860
0.25
0.2
0.15
0.1
0.05
0
0h
48h-ET
96h-ET

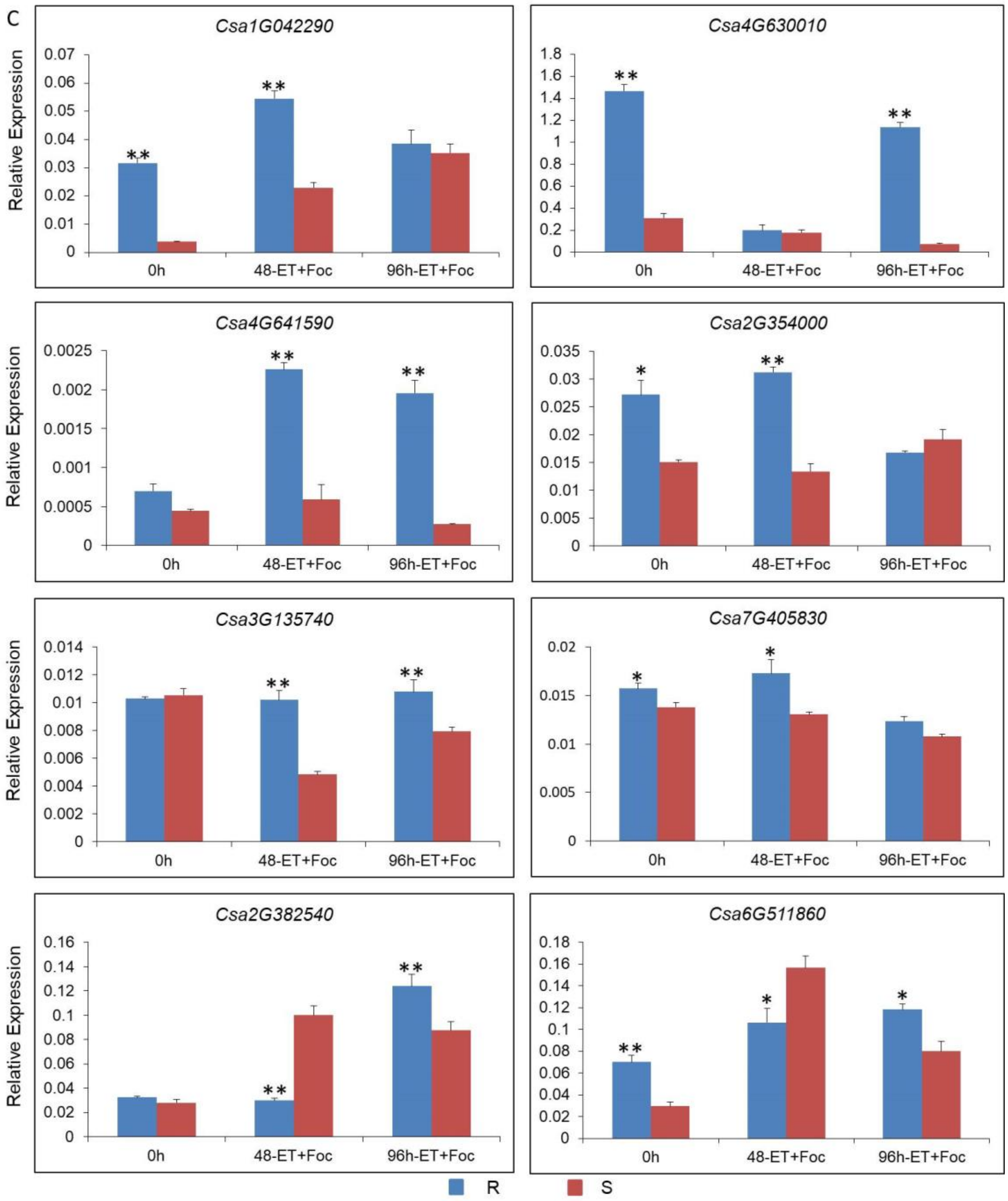

Additional file 3: Figure S2. Expression profiles of unselected candidate genes. The expression profiles showed a high relative expression level in cucumber seedlings at 0, 48, and 96 h after (A) inoculation with *Fusarium oxysporum* f. sp. *cucumerinum* (Foc), (B) spray treatment with exogenous ET, and (C) after inoculation with Foc and spray treatment with exogenous ET. R, 'Rijiecheng' (Foc-resistant line); S, 'Superina' (Foc-sensitive line).
