# Supplementary material for: Transcriptome analysis reveals ethylene-mediated defense responses to Fusarium oxysporum f. sp. cucumerinum infection in Cucumis sativus L
Source: BMC Plant Biol. 2020 Jul 16;20:334. doi: 10.1186/s12870-020-02537-7 (PMC7364617; doi:10.1186/s12870-020-02537-7)
Supplement: Supplementary file 4 — Additional file 4: Table S2. Primer sequences used for ethylene- related differentially expressed genes. [file 12870_2020_2537_MOESM4_ESM.pdf]

Additional file 4: Table S2. Primer sequences used for ethylene- related differentially expressed genes.

Gene ID	The Primer Sequences
Csa4G630010	CCACGTAAGAAGACTAGG
	GTTGCTGTGGTTATTGTTA
Csa3G018320	TGGTGGTTAAGAAAGAGAA
	CGGCATATCCTTCAAGTA
Csa7G049230	AAGGCTAAAACCAACTTTC
	GGTAGGACTAGGACTTTG
Csa1G042290	TGCTGGAATACATCTGTAA
	CGTTGGATAACTGATCGA
Csa7G405830	GTCTGCATTCTTTCCTTATAG
	CACGATACAATAGCACAAG
Csa2G356600	CCGGACCTGATTTTACAA
	CGGAGAGAAGTTTGATGA
Csa2G010390	AGGAAGTATTATGATGATATTGG
	GATGTCTTGTGGGTTGTA
Csa7G318990	GGTATAATGCTTCACTTCAA
	ATTAGATGTCTCTGCTGTT
Csa3G135690	GATACCATCCTCACTCAA
	TTCTCCAACATTCTCCAT
Csa6G160180	TACTGAACCACGGAATAGA

	GAGATGACGGAGGAAGAA
Csa7G049230	CATTCATTCAACCTCAGT
	ACCGTATAGGATAACCAT
Csa6G318160	ATAGCGAGGAGCAACAAG
	ATCATCCAAACATCCAAATCAA
Csa1G042290	GGAGAATTACAGCAGTGAA
	GAAGATGGTGAAGGATGAG
Csa4G630010	TCAATACGGCAGAAGAAG
	TTGCTGTGGTTATTGTTATC
Csa4G641590	ATCAGAATCATCTCCAACAACA
	ATTTCCGCAACCCACTTT
Csa2G354000	AGTGATAATGAGGATAGTAACAAC
	GAAGCAGAGTGAAGAGAATG
Csa3G135740	AACGAGTGATGGAGATTG
	GTAGTAGTGGCATACAAGT
Csa7G405830	ATGATGGAGTCCTGTGAT
	TAAGCAAGAGCGATTAGC
Csa2G382540	GGATTGTGAGGAGCCATT
	TTCCGCCGTAATCAAGTAG
Csa6G511860	TGGTGGTCTTATTCTTCT
	TGATAACAATGGAGTGATG
